# On the phenomena of partial crystallization of highly undercooled magnesium silicate molten droplets

**DOI:** 10.1038/s41598-021-93135-6

**Published:** 2021-07-01

**Authors:** Ganesh Shete, Sushil Mishra, Shyamprasad Karagadde, Atul Srivastava

**Affiliations:** Department of Mechanical Engineering, Indian Institute of Technology Bombay, Powai, Mumbai, 400076 India

**Keywords:** Early solar system, Techniques and instrumentation

## Abstract

The present work reports real-time observations of the phenomena of partial crystallization of one of the glass-forming materials, namely enstatite (MgSiO_3_) from its supercooled liquid droplet. Initially, the molten droplet has been held under purely non-contact conditions using the aerodynamic levitation technique. The desired levels of undercooling have been achieved by deliberately making the levitated molten droplet touch a thin molybdenum wire and hence to initiate heterogeneous nucleation from the point of contact. Influence of thermal parameters like undercooling, cooling rates and recalescence on the process of crystallization is investigated. To understand and report the morphological properties and extent of crystallinity, the solidified enstatite samples have been characterized using optical/scanning electron microscopy (SEM) and X-ray diffraction (XRD) respectively, which confirmed the formation of partially crystallized enstatite spherules and fully glass spherules. XRD showed sharp peaks of enstatite, which confirm crystallinity and a halo profile confirms the amorphous phase of enstatite. Based on the observations of several experiments, we propose the effect of thermal parameters such as levels of undercooling and recalescence on the partial crystallization, as well as partial glass formation from the initially molten droplets of enstatite composition.

## Introduction

Enstatite is one of the widely explored silicate materials and is believed to be a potential candidate for a range of applications, such as biomedical (dental implants, bone substitutions)^1^, opto-electronic (laser sources, optical fibre amplifiers)^2^ and applied sciences (geophysics, earth and planetary sciences)^3^. Developing a fundamental understanding of the nucleation of enstatite is an important step towards the study of the process of its crystallization. Ray et al*.*^4^ studied internal and external crystallization on lithium silicate glassy samples and observed the process of partial crystallization. Pablo et al*.*^5^ carried out partial crystallization experiments of phosphate glasses and studied the structural and luminescence properties using SEM, XRD and Raman spectroscopy. Massera et al*.*^6^ studied the thermal history and partial to full crystallization on glass reactivity on three phosphate bioactive glasses and suggested that only one parameter is insufficient to study the complex mechanism of crystallization. Several authors, as described in the above literatures have studied the process of partial crystallization of various glass-forming materials and glass–ceramics. The phenomenon of partial crystallization has attracted the interest of the scientific community since it holds importance in a range of scientific and technological applications.

However, the heterogeneous nucleation offered by contaminants such as container surface prevents achieving desired levels of undercooling. Therefore, numerous authors have studied the processes such as crystallization and glass formation using different levitation techniques that offer an uncontaminated and undisturbed atmosphere. Hamai et al*.*^7^ studied both heterogeneous and homogeneous crystallization using magnetic levitation. Motokawa et al*.*^8^ studied crystallization and perfect spherical glass formation under levitated conditions. Li et al*.*^9^ studied multiple site crystallization using high-speed videography in an undercooled mullite melt using aero-acoustic levitation. Yasutomo et al*.*^10^ prepared spherical optical crystals that are useful for ball lenses by levitation methods. Lü et al*.*^11^ investigated the solidification of undercooled metallic alloy by employing electromagnetic levitation. Cao et al*.*^12^ studied crystallization of various samples using aero-acoustic levitation.

It can be seen from the above literature that various levitation methods such as magnetic levitation, acoustic levitation have been widely explored to study crystallization or glass formation. However, such containerless techniques of levitation strictly depend on conducting or magnetic properties of materials under consideration. In this direction, the aerodynamic levitation methodology, which is independent of the properties of the material under consideration, becomes important as it also allows control over the possible initiation of heterogeneous nucleation of any desired level of undercooling by deliberately making the molten sample touch an external nucleating agent. In the context of aerodynamic levitation based crystallization experiments on magnesium silicates, Beitz et al*.*^13^ attempted to create accretionary rims around the chondrule like objects at room temperature 20 °C and at 1100 °C and tested the hypothesis that these rims were formed in the solar nebula by the accretion of dust on surfaces of chondrules. Mishra et al*.*^14^ reported an experimental study of crystallization of magnesium silicates under aerodynamically levitated conditions. Pack et al*.*^15^ developed an in-house aerodynamic levitation facility equipped with inductively coupled mass spectrometry (ICPMS) to perform evaporation and reduction experiments on silicates to study chemical analysis.

Experimental studies based on partial crystallization of glass-forming materials under container-less conditions are highly scarce. The primary motivation of performing such levitation-based experiments, particularly on magnesium silicates (enstatite), has been motivated by its importance in the field of earth and planetary science. Enstatite is believed to be a part of chondrules in chondritic meteorites, which, in turn, is the remnant of the early solar system^3,16–18^. These chondrules are considered to be formed from silicates (crystalline and glassy) under freely floating conditions^19^. Several authors suggested that chondrules were formed under nearly equilibrium conditions and hence employed very slow cooling rates using sample holding techniques^20^. The sample holding technique offered immediate heterogeneous nucleation sites and hence observed lesser levels of undercooling^21–24^. Faure et al*.*^22^ studied olivine morphology as a function of slower cooling rates and lower undercooling levels since the use of electric furnaces offer relatively small cooling rates. But some of the authors have employed relatively higher cooling rates as well^25–28^.

The discussion of cooling rates that are applied to cool the silicate samples from high temperatures can be further extended to levels of undercooling obtained. Nelson et al*.*^28^ prepared glassy crystals of enstatite spherules (which were strikingly similar to chondrule-like objects) from rapid crystallization but undercooling levels achieved were restricted to 400 K. Their experimental conditions resemble the conditions of chondrule formation by small-scale thermal events(like volcanism). Shete et al*.*^29^ studied the morphological transition of silicate crystals obtained from highly undercooled melt droplets under aerodynamically levitated conditions for various levels of undercooling. Nagashima et al*.*^30^ performed levitation experiments on enstatite spheres (diameter < 3 mm) and proposed an undercooling regime of ΔT ~ 260–860 K for crystallization to occur since beyond this regime crystallization is not possible due to glass formation. Tangeman et al*.*^31^ studied the crystallization and glass formation at a relatively higher cooling rate for magnesium silicate (forsterite) at weight proportions such as 3.5 and 40 mg that produced glassy and crystalline silicate spherule respectively. The process of partial crystallization (or the extent of partial zones) depends heavily on the levels of undercooling, recalescence and cooling rate. Such a study considering pure enstatite as the starting material, that has a range of applications, has not been attempted in the past, as per the best of the knowledge of the authors. In view of this, the current study focuses on the heterogeneous crystallization of aerodynamically levitated and deeply undercooled enstatite molten spherules subjected to relatively higher cooling rates.

Nagashima et al*.*^32^ performed crystallization experiments on enstatite spherules hung by a thin wire of Pt–Rh (sample holding technique) under normal gravity conditions and found completely crystalline enstatite but didn’t find glassy parts of enstatite. But under microgravity conditions, out of 14 enstatite samples, only two samples showed the combination of both crystalline and glassy parts of enstatite spherules. This is probably because enstatite samples had a larger area of contact (due to the sample holding technique) for nucleation and subsequent crystallization. A similar set of results have been compared, obtained and reported in the current study where using the levitation technique, purely non-contact conditions have been simulated but heterogeneous nucleation on these levitated enstatite samples has been triggered by a thin wire of molybdenum. The clear demarcation between the experimental methodology employed by Nagashima et al*.*^32^ and that considered in the present study is that the authors^32^ performed microgravity experiments using a sample holder, on the other hand, the present experiments correspond to containerless experiments under normal gravity conditions. Also, nucleation in^32^ was initiated along the contact area of the sample holder (which offered mostly complete crystallization) whereas in the present experimental work, nucleation has been initiated using a thin wire of molybdenum offering conditions that are conducive majorly for partial crystallization at lower undercooling values. By virtue of this arrangement, the enstatite samples in the present levitation-based study did not have a large area of contact for nucleation, as that in the case of the sample holding experiments reported earlier by Nagashima et al*.*^32^. This also suggests that such a small contact with solidifying spherule offers the possibility of various levels of recalescence and cooling rate, which, in turn, can alter the amount of crystalline and glassy zones. A similar set of experiments where an external nucleation site was created by using a thin wire of molybdenum have been reported in^33,34^ for semiconductor sample materials (such as Si, Si-1 at%Sn). However, the application areas targeted in these studies^33,34^ are different than that of the present study wherein enstatite has been considered as sample material due to its significance in the context of earth and planetary sciences, in particular towards developing an understanding of formation mechanism of the early solar system.

Richet et al*.*^35^ found out glass transition temperature of enstatite to be approximately 750 °C. In the context of heterogeneous experiments performed in the current study, a range of temperatures for heterogeneous nucleation (triggered using a thin wire of molybdenum) was selected between the enstatite melting point temperature and its glass transition. Maeda et al*.*^36^ hypothesized that metals that have higher melting points should be preferred as a tool to initiate (heterogeneous) nucleation and hence employed molybdenum to crystallize enstatite which has application in glass–ceramics. For this reason, a thin wire of molybdenum has been employed to externally nucleate the initially molten enstatite spherule in between its melting point and glass transition temperature. Possible influence of thermal parameters like undercooling, cooling rate and recalescence has been revealed using a cooling curve. Solidified enstatite samples obtained after experimentation have been observed using an optical microscope and showed the combination of both glassy and crystalline parts of enstatite as that reported in the literature^32^.

The possibility of enstatite to partially crystallize under heavily undercooled conditions has not been investigated to the best of the knowledge of the authors. Also, there are lacunae in the literature related to the study of nucleation and crystallization of enstatite spherules when they are deeply undercooled. This study, equipped with in situ high-speed imaging techniques, is expected to provide a pathway for the kinetics of crystallization. Solidification experiments have been performed for various levels of deeper undercooling obtained by employing an aerodynamic levitation facility. Plausible effects of thermal parameters (obtained from the cooling curve of crystallization) such as levels of undercooling, cooling rate and recalescence on the phenomenon of partial crystallization in terms of the extent of different zones (crystalline and glassy) have been discussed. Partially crystallized spherules obtained after experiments and their formation conditions have been discussed and compared with some of the experimental works available in the open literature*.* Solidified enstatite samples obtained after experimentation have been observed under optical/scanning electron microscopy (SEM) and the crystallinity of these samples has been confirmed by X-ray diffraction (XRD). XRD analysis has also been performed on the powdered form of all the solidified samples to ascertain any possible internal crystallization.

## Experimental setup

To elucidate the impact of thermal parameters on possible crystallization of initially molten enstatite spherules, a series of experiments were performed using aerodynamic levitation experimental facility. Details of the experimental facility have been recently reported elsewhere^29^. In brief, aerodynamic levitation offers purely non-contact experimental conditions leading to the realization of relatively higher levels of undercooling before the molten droplet undergoes solidification. Ensuring the highest levels of controls over the operating parameters, it is possible to cool the initially molten droplet much below its glass transition temperature leading to its complete transformation into its amorphous form. In addition, while complete glass formation is possible, one can also achieve heterogeneous nucleation at any desired level of undercooling by bringing the levitated molten droplet in physical contact with suitable external nucleation triggering surface (e.g. nozzle surface, solid dust particles, a thin metallic wire, etc.). This, in turn, offers better control on undercooling levels as well. In the context of the experiments reported in the present study, heterogeneous nucleation of the levitated enstatite molten droplets has been triggered using a thin molybdenum wire (melting point = 2623 °C, diameter = 150 µm). To study the effect of level of undercooling on the processes of complete crystallization, partial crystallization and glass formation, the physical contact between the molten droplet and the molybdenum wire has been established at various stages of the cooling curve of the molten droplet i.e. between its melting point temperature (T_m_) and the glass transition temperature (T_g_).

**Figure 1:**
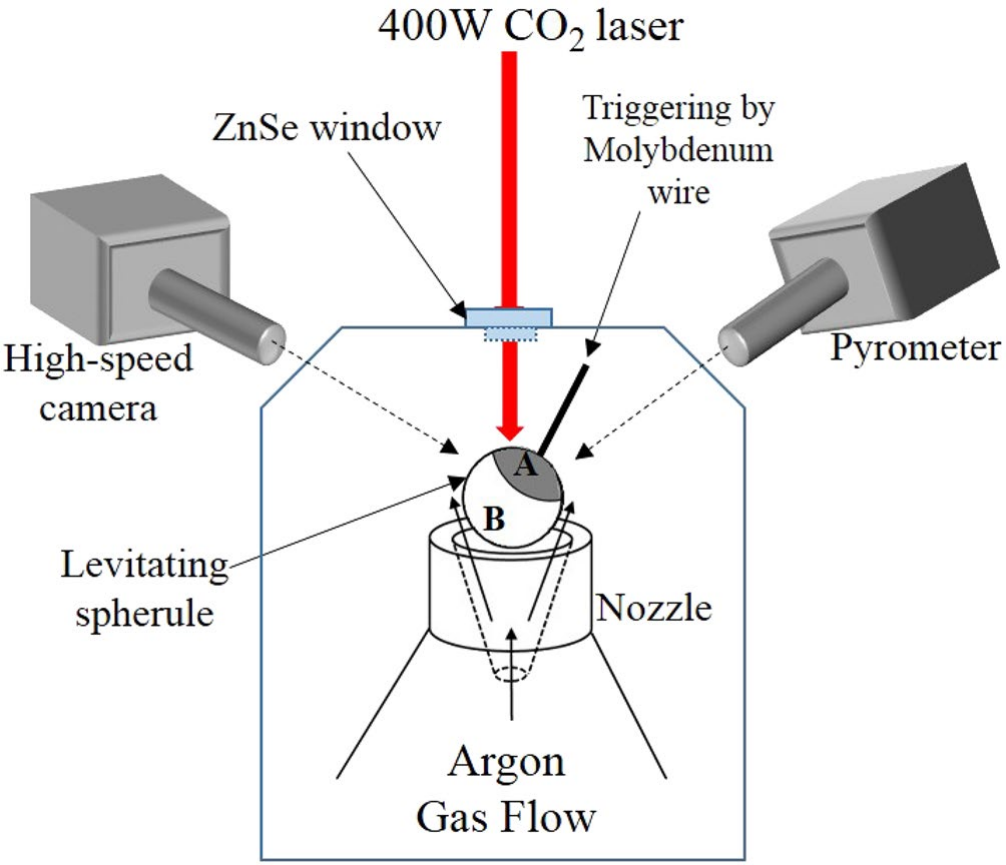
A schematic arrangement which shows levitating enstatite spherule where crystalline part (region A) nucleates and subsequently crystallizes due to triggering by a thin wire of molybdenum while remaining enstatite turns into a glass (region B) (drawing prepared on MS Office (2016) platform).

**Figure 2:**
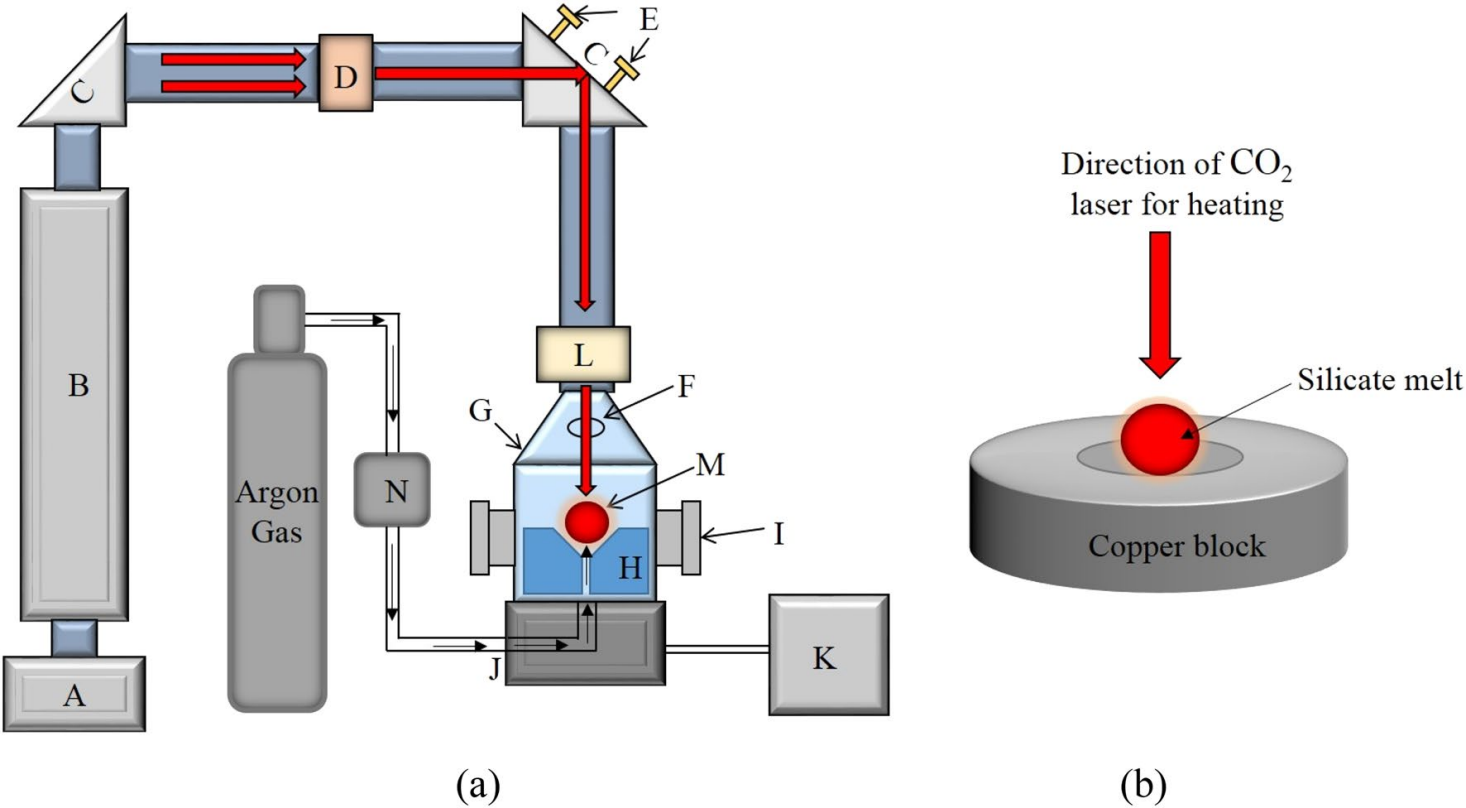
(**a**) Schematic of the complete experimental setup showing important devices. A-Laser power supply (Synrad i401), B-Laser head, C-Mirror, D-Lens assembly, E-Laser beam focusing screws, F-Pyrometer ports, G-Process chamber, H-Nozzle, I-Optical ports, J-Gas port, K-Chiller, L-Zn-Se window, M-Levitating spherule, N-Mass flow controller. (**b**) Schematic diagram showing the method of preparation of silicate spherules, consisting of a copper block hearth (drawing prepared on MS Office (2016) platform).

The schematic representation of the physical configuration of an aerodynamically levitated molten droplet with the provision of externally induced heterogeneous nucleation using a molybdenum wire is shown in Fig. 1. The starting material employed is enstatite (MgSiO_3_) powder with 99.99% purity (*Goodfellow*: Supplier of materials for research and development). Enstatite spherules of ~ 2.5 mm diameter were prepared using a laser hearth (equipped with an experimental facility) (see Fig. 2b) and levitated using a conical nozzle made up of aluminum (see Fig. 2a).

As shown in (Fig. 2a), argon gas with a flow rate of ~ 400 ml/min has been employed for achieving aerodynamic levitation. Initially solid spherules were completely melted using a high power CO_2_ laser (*Firestar i400*, λ = 10.6 µm and spot size = 1 mm, the maximum power output of 400 W). The molten state of the enstatite spherules was ensured by heating the sample well above its melting point temperature (T_m_ = 1560 °C)^32^. Moderate to rapid cooling rates were achieved by suitably controlling and/or switching off (for quenching) the laser power. Real-time visualization of the phenomena of solidification (partial crystallization and vitrification) has been achieved by integrating the experimental facility with CMOS based high-speed camera (Phantom Digital High-speed VEO410L). Images have been recorded at 4000 frames/s with a spatial resolution of 640 × 480 pixels. The time history of the surface temperature of the solidifying droplet has been recorded using an optical pyrometer whose default acquisition rate is 10 Hz. The pyrometer is of Chino-IR-CAS series having measurement range 600–3000 °C with an emissivity value of *ɛ* = 0.9 at the working wavelength (0.9 µm). This emissivity value corresponds to materials such as magnesium silicate alloys, as employed in the present set of experiments as model material (Enstatite)^37^. The operating wavelength of pyrometer is 900 nm and accordingly, appropriate emissivity corrections have been applied to maintain uniformity in the measured values of temperature over the design range. With such emissivity corrections, the error in temperature measurement is well within acceptable limits. For the temperature range involved in the present set of experiments, an accuracy rating of ± 0.5% of the measured value of temperature is achieved with the employed pyrometer. In addition to this, major findings of the present experiments are focussed majorly on the effect of two thermal parameters, namely undercooling and amount of recalescence on the crystalline and glassy zones. These parameters are primarily the differences in two temperature values, and hence the errors are almost negligible, w.r.t. to the orders of magnitude of high undercooling levels achieved. The phenomenon of heterogeneously induced onset of nucleation, once the molybdenum wire physically touches the molten droplet, has been captured in the form of the instantaneous sharp increase in the surface temperature of the solidifying sample (through optical pyrometer) as well as a sudden increase in the intensity levels of the two-dimensional images as a result of the release of the latent heat of crystallization (recalescence). In contrast, no such sudden change (increase) in the instantaneous temperature and/or intensity levels are to be expected in the case wherein the solidifying spherule gets completely transformed into a glass (achieves temperatures that are well below the glass transition temperature of enstatite).

Solidified spherules of enstatite obtained after experimentation were further characterized by an optical microscope and SEM. To verify the crystallinity of the solidified enstatite samples, X-ray diffraction (XRD)measurements were performed on Empyrean diffractometer (*λ* = 1.54 Å) having variable spot focus capability. XRD analysis for the powdered form of enstatite samples have been performed with spot focus capability of 2 mm while that on the small but crystallized surface of spherules have been performed with 0.5 mm. XRD profiles performed on the crystallized surface of solidified enstatite samples offered the qualitative results of crystallinity of the solidified enstatite samples, while that performed on the powdered form of samples offered information about any possible internal crystallization. A thin gold–palladium coating has been applied on the enstatite spherule, which got solidified completely to avoid charge build-up. This avoids the charging of electrons since enstatite is non-conductive. The sample is then further observed under the SEM (scanning electron microscope).

## Results and discussion

Experimental observations pertaining to partial crystallization and partial vitrification of levitated enstatite molten droplets have been discussed in this section. The range of undercooling levels achieved in the experiments has been achieved by choosing the undercooled melt temperature at which the levitated molten droplet of enstatite is made to touch the thin molybdenum wire. For ready reference and ease of discussion, solidified samples have been termed as A, B, C, …., H.

Samples A, B, C and D are characterized by relatively lower levels of undercooling (between the range of ΔT ~ 400–525 K) while samples E, F and G were offered higher undercooling levels (ΔT > 615 K). For samples A to D, a detectable phenomenon of recalescence, as recorded using optical pyrometer as well as high-speed camera, could be clearly observed which indicated towards the crystallization of the initially molten droplet as the heterogeneous nucleation was triggered by molybdenum wire. However, samples E, F and G, wherein the physical contact between the molten sample and the molybdenum wire was established at much deeper levels of undercooling (closer to the glass transition temperature of enstatite), crystallization was observed for samples E and sample G where clear recalescence was observed in the cooling curve while for sample F detectable recalescence was absent. Sample H corresponds to the extreme case of vitrification wherein no attempt of heterogeneous nucleation was made and the molten droplet, under purely non-contact conditions, was allowed to cool down to temperatures well below the glass transition temperature. This highly undercooled sample got completely transformed into glass. The crystallized and/or glassy nature of the resultant solidified samples has been ascertained through both surface and powder XRD analysis in each case.

It is worth clarifying here that the undercooling levels achieved in the present experimental work are well above the hypercooling limit (which is thermodynamically defined as Δ*T*_*hyp*_ = Δ*H*_*f*_*/C*_*p*_ where Δ*H*_*f*_ is the enthalpy of crystallization and *C*_*p*_ is the specific heat of liquid). The value of hypercooling limit Δ*T*_*hyp*_ for enstatite melt is close to 507 *°*C^32^. Beyond this limit, the solidification process changes drastically. When Δ*T* < Δ*T*_*hyp*_, the liquid (present after the recalescence) is solidified when the recalescence is over. But when Δ*T* > Δ*T*_*hyp*_, the molten sample solidifies completely in the recalescence. To our understanding, such an experimental work on enstatite as sample material has not yet been addressed (with undercooling greater than hypercooling limit) in the open literature that endorses the novelty of the present work.

### Observations of surface features for smaller crystalline zone

**Figure 3:**
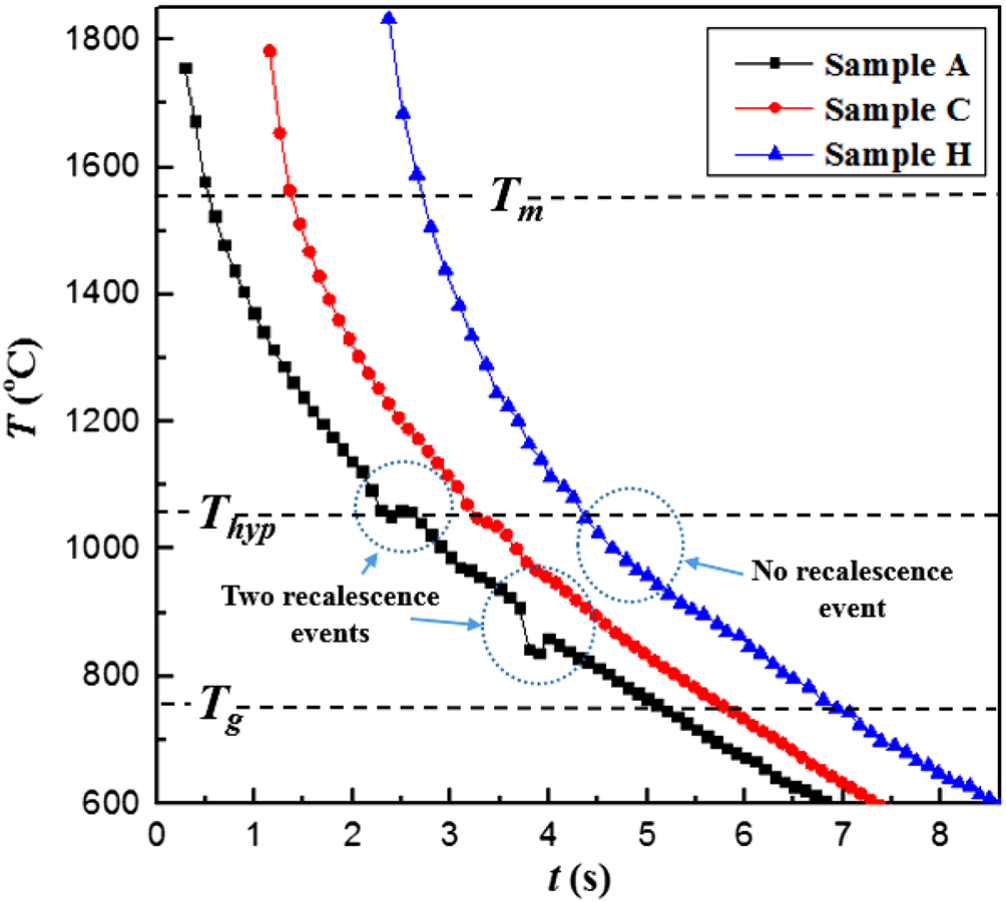
Cooling curves for enstatite melt sample A (ΔT = 510 K) and sample C (ΔT = 525 K) under a levitated condition which shows recalescence events on the cooling curve hence indicates the presence of nucleation and subsequent crystallization while sample H doesn’t exhibit recalescence indicating a process of vitrification. Melting point temperature (T_m_), hypercooling temperature (T_hyp_), and glass transition temperature (T_g_) of enstatite is shown by a broken line.

Solidification patterns of samples A, C and H have been discussed in this section. The temperature vs time history of these samples, as recorded using optical pyrometer, has been shown in Fig. 3 which shows clear demarcations between different cooling curves in which onset of nucleation occurs at various temperatures.

An otherwise monotonically decreasing temperature of the molten droplet shows the signs of recalescence (instantaneous increase in temperature) due to the onset of heterogeneous nucleation as the thin molybdenum wire is made to touch the levitated molten droplet. The sudden release of latent heat of crystallization results in instantaneous heating of the solidifying front, a phenomenon which was ascertained through the cooling curve. For instance, the two points of recalescence events seen in Fig. 3 for sample A corresponds to two instantaneous heating rates of 90 and 240 °C/s. The cooling curves for samples C and H have also been shown in Fig. 3. Sample C does not exhibit any detectable recalescence phenomenon but does show a slight deviation in the cooling curve near hypercooling temperature line (T_hyp_). The cooling curve for sample H shown clearly shows a complete absence of any recalescence event as the molten droplet is cooled down while being in purely non-contact conditions. The temperature of the solidifying sample monotonically decreases, goes below the glass transition temperature and undergoes complete vitrification leading to glass formation.

**Figure 4:**
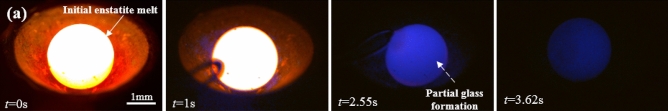
High-speed camera images of enstatite melt sample A where bright part at time instant t = 0 s indicates completely molten initial enstatite sample, at t = 1 s shows drop in the temperature while cooling and also at the same instant molybdenum wire comes in contact with the sample, at t = 2.55 s shows initial nucleation and t = 3.62 s shows final partly glassy and partly crystalline enstatite sample. Scale bar in the first image applies to all subsequent images.

**Figure 5:**
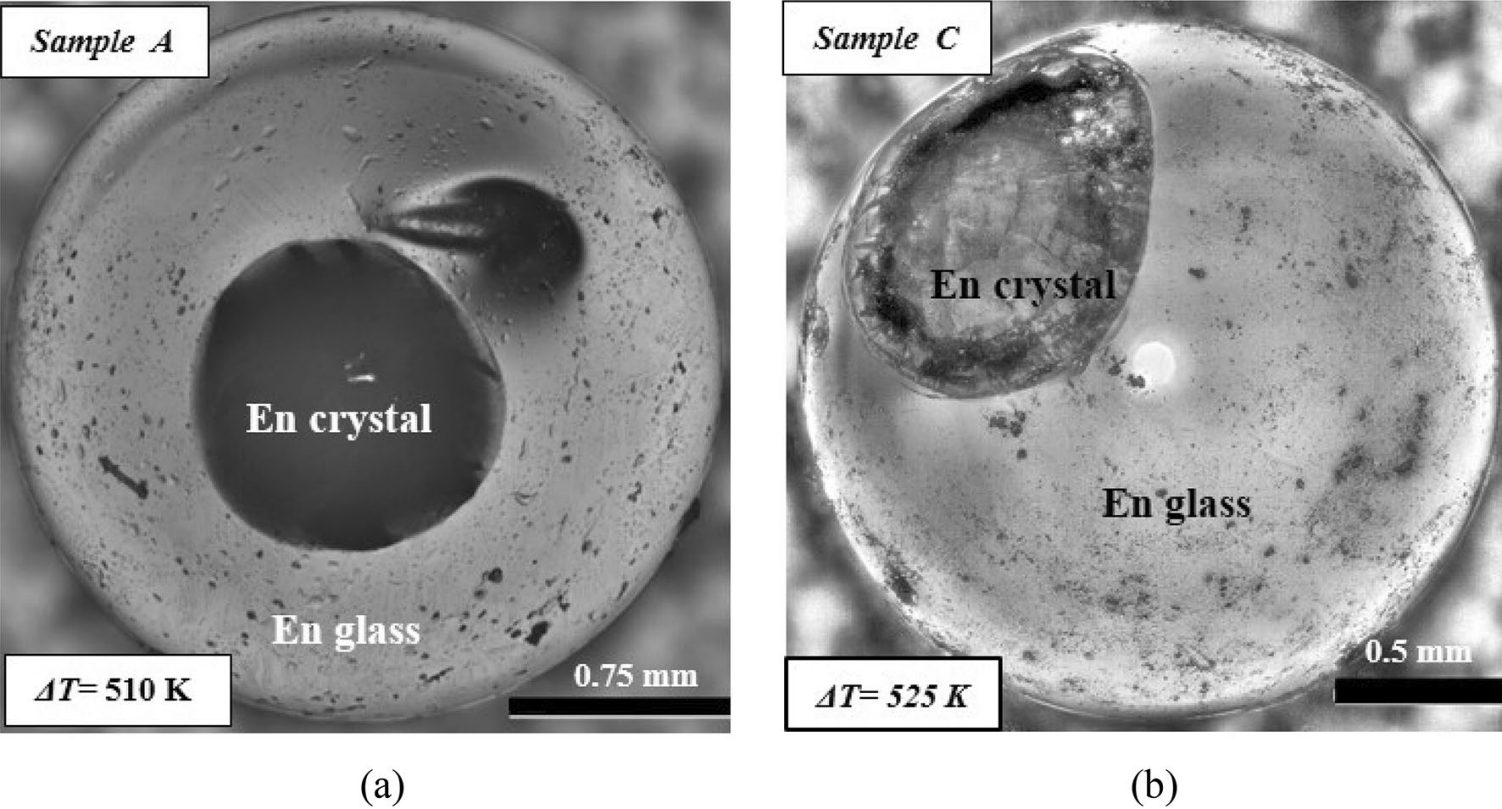
(**a**) Optical microscope images of the solidified enstatite (**a**) sample A (ΔT = 510 K) (**b**) sample C, (ΔT = 525 K) both processed under levitated conditions and triggered by a wire of molybdenum.

Figure 4 shows the time-lapsed high-speed images of the molybdenum wire-induced onset of nucleation for sample A. The images corresponding to t = 0 s show the initial molten droplet under complete non-contact conditions (aerodynamically levitated). As time proceeds further, the crystallizing front created due to the molybdenum wire which advances in the bulk of molten droplet can be clearly seen on the sample surface. The onset of heterogeneous nucleation, triggered using a thin wire of molybdenum (at t = 1 s) leads to the realization of partial glass formation and partial crystallization in sample A. It was highly expected that a very small crystallizing zone could have been formed since the enstatite sample experiences a very small amount of recalescence event, which has been further confirmed by optical microscopy. The clear demarcation between the partial crystal (formed due to liberation of latent heat) and partial glass formed while cooling the enstatite sample can be seen using the employed high-speed imaging technique. These high-speed based imaging observations have been supported through optical micrographs of the final solidified enstatite sample A. These optical micrographs have been shown in Fig. 5.

Figure 5a depicts the crystallization behavior of sample A (En crystal and En glass can be seen), which followed the thermal history shown in Fig. 3. Since enstatite is rich in silica, as the temperature drops, molecular mobility decreases which subsequently increases the melt viscosity and hence inhibits further nucleation and crystal growth. The crystalline phases that evolved during the process have been identified through XRD phase analysis (see Fig. 11a) performed on the crystalline part of the spherule. These XRD analyses clearly showed the peaks of enstatite. This suggests that both thermal history as well as the XRD analysis, ascertain the crystallinity.

Similar observations are also to be made for sample C as shown in Fig. 5b along with its XRD profile (Fig. 11a). The smaller volume of enstatite crystalline region than enstatite glassy region for both samples A and C is attributed to the fact that the recalescence event/s as shown in the thermal history of these samples are lesser as compared to the all samples studied in the next subsection. The discussion about the thermal history has been corroborated by using optical microscopy and the XRD method. Figure 5b shows the difference between enstatite crystal and enstatite glass of sample C. XRD profile also shows some major sharp peaks which confirms the crystallinity of the enstatite crystalline part of sample C (Fig. 11a).

**Figure 6:**
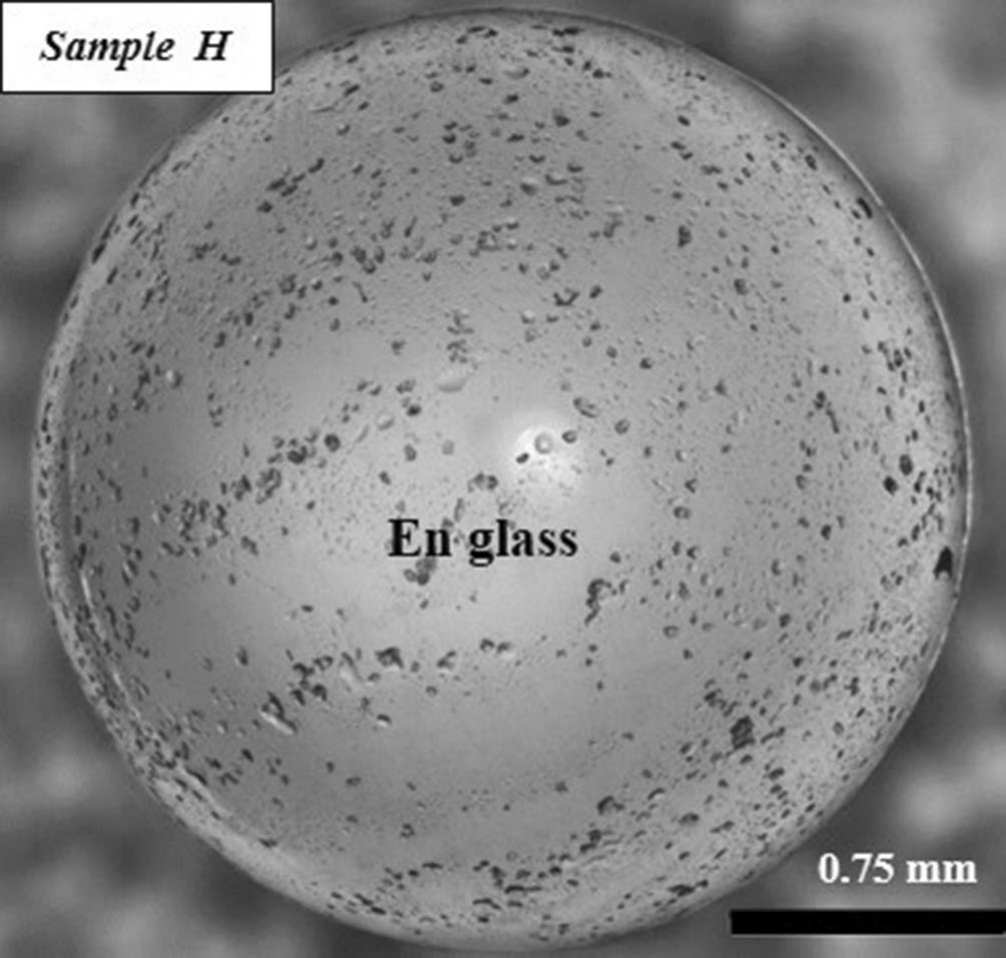
Optical microscope images of the solidified enstatite sample H both processed under levitated conditions and not triggered by a wire of molybdenum.

Figure 6 shows the optical microscope image of the solidified sample H where only the glassy part can be observed and no crystalline part is to be seen unlike in the case of samples A and C discussed earlier. Figure 11a does show an initial amorphous halo and neither major nor any minor peaks are to be seen, thus confirming the process of vitrification of the initially molten enstatite spherules.

Similar approach and set of results have also been reported by Nagashima et al.^32^ where enstatite melts were held using metallic wires or rods. The authors in^32^ observed that nucleation always took place from these wires and/or rods and obtained complete crystallization under normal gravity conditions while partial crystallization under microgravity conditions. To the best of the knowledge of the authors, this is one of the few attempts to reproduce such mixed structures (crystalline and glassy) under levitated and normal gravity conditions. In the context of chondrule crystallization study, the plausible mechanism that can be explained which led to the formation of such mixed structures for enstatite (magnesium silicates) can be either due to smaller recalescence events under levitated conditions (as per the present study) or microgravity conditions (reported by Nagashima).

### Observations of surface features for larger crystalline zone

**Figure 7:**
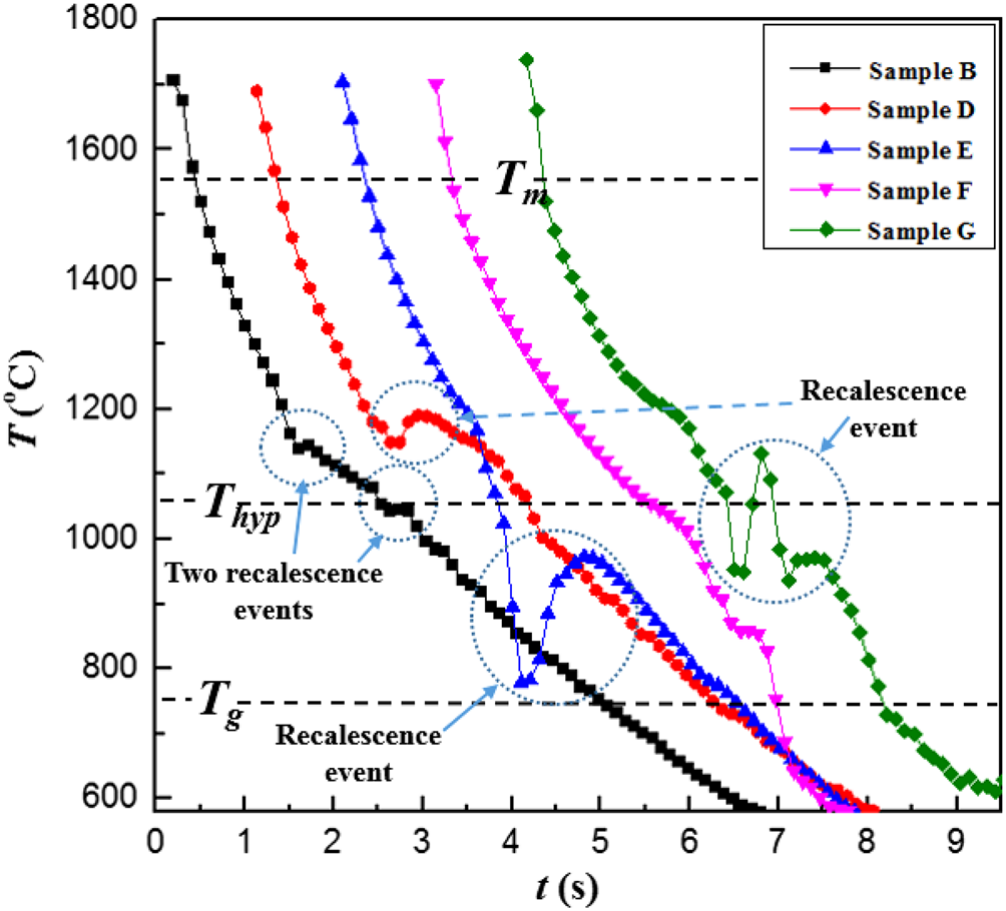
Cooling curves for enstatite melt sample B (ΔT = 519 K), sample D (ΔT = 411 °C), sample E (ΔT = 782 K), sample F (ΔT = 702 K) and sample G (ΔT  = 611 K) under a levitated condition which show recalescence events on cooling curve hence indicates the presence of nucleation and subsequent crystallization. Melting point temperature (T_m_), hypercooling temperature (T_hyp_), and glass transition temperature (T_g_) of enstatite is shown by a broken line.

In this subsection, the solidification phenomena of samples such as B, D, E, F and G that observed a larger crystalline zone relative to the glassy zone have been discussed. Sample B shows two recalescence events, first quite above the hypercooling temperature line (T_hyp_) and second just near T_hyp_ line contrary to the cooling curve of sample A where second recalescence was approaching the glass transition line (Fig. 7). Therefore, a larger crystalline zone has been observed for sample B as compared to sample A. It is to be noted sample D has experienced a relatively lesser undercooling level (ΔT ~ 410 °C) before the molten droplet was made to heterogeneously nucleate (Fig. 7). The instant of onset of crystallization of sample D is evident through the realization of the recalescence event due to which a sudden rise in temperature of the solidifying droplet (by about 45 °C) takes place before the sample resumes the cooling curve. Compared to sample A, B and C, sample D exhibits the strongest recalescence phenomenon well above the hypercooling temperature line, hence it is expected that the extent of crystallization achieved in D would be relatively higher than that in the other three samples.

**Figure 8:**
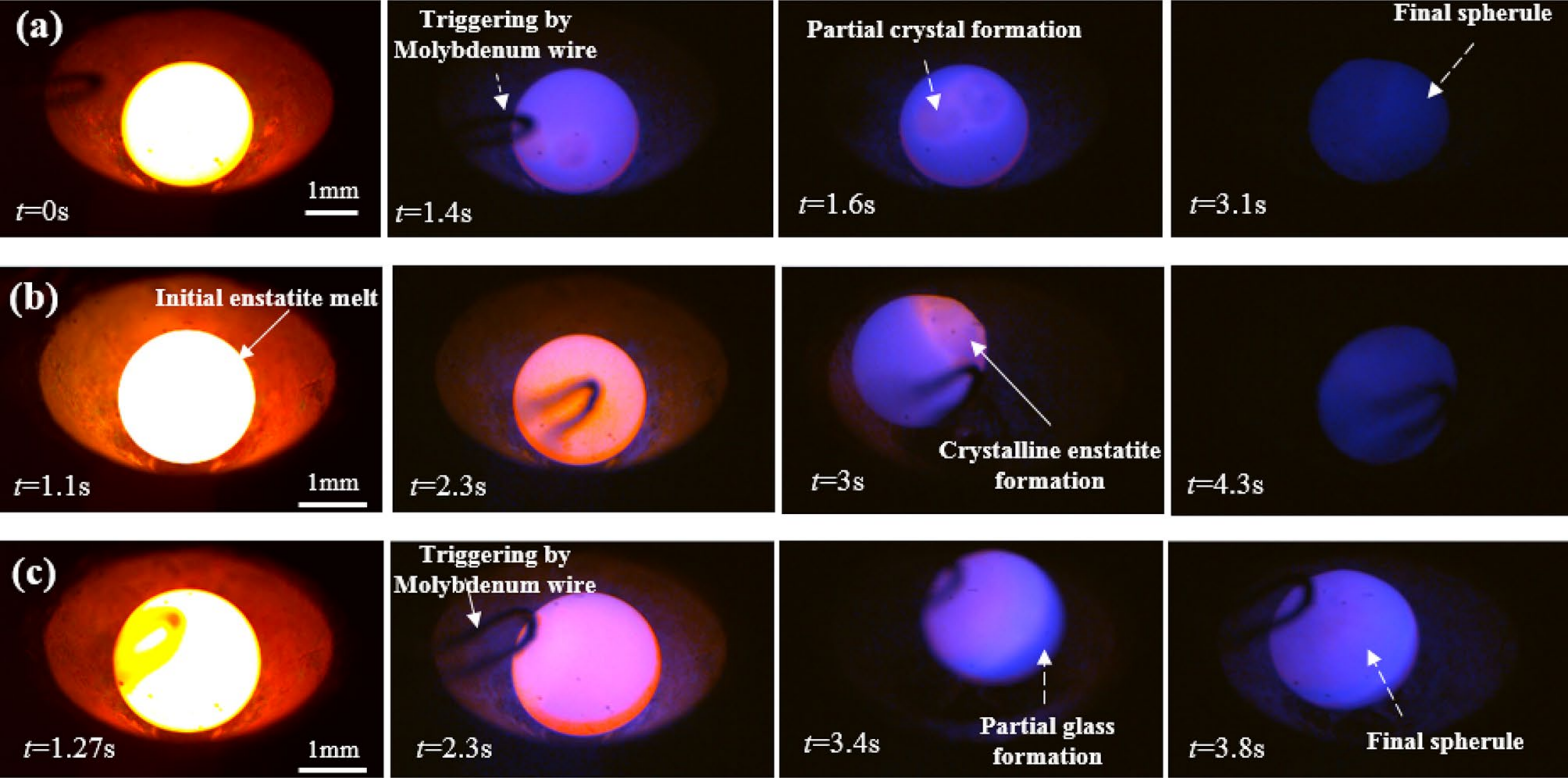
High-speed camera images of enstatite melt. (**a**) Sample B also shows bright part at time instant t = 0 s indicates completely molten sample, at t = 1.4 s shows initial nucleation and drop in temperature while cooling and t = 1.6 s shows partly glassy and partly crystalline enstatite and t = 3.1 s shows finally solidified sample. The scale bar in the first image applies to all subsequent images. (**b**) Sample E where bright part at time instant t = 1.1 s indicates completely molten sample, at t = 2.3 s shows drop in the temperature while cooling, at t = 3 s shows wire touching sample and t = 4.3 s shows final partly glassy and partly crystalline enstatite sample. (**c**) Sample G also shows bright part at time instant t = 1.27 s indicates completely molten sample, at t = 2.3 s shows initial nucleation and drop in temperature while cooling and t = 3.4 s shows partly glassy and partly crystalline enstatite and t = 3.8 s shows finally solidified sample.

The temperature of sample E reached near to the glass transition temperature of enstatite which is well below the hypercooling temperature line (T_g_ = 750 °C) hence it experienced the largest undercooling level (ΔT = 782 °C) as compared to all other samples. The cooling curve of sample E shows clear deep till the temperature reaches near the glass transition temperature (Fig. 7). As it approaches the glass transition temperature of enstatite (T_g_ = 750 °C), a sudden sharp jump in the temperature profile is to be seen which again raises the temperature to ~ 1000 °C. This sudden and significantly large temperature rise (by about 195 °C) has been confirmed to be the result of recalescence phenomenon as observed by high-speed imaging technique and XRD analysis, an aspect of the work that has been discussed further. Figure 7 also shows the cooling curves for samples F and G. As compared to sample E and G, the heterogeneous nucleation in sample F was triggered at a temperature that was very close to the glass transition temperature of enstatite (well below T_hyp_ line) and did not observe any detectable recalescence. As the triggering is done near the glass transition, it is reasonable to expect the most of the sample material gets converted into its glassy phase, thereby leaving a very little possibility of the sample getting converted into the crystalline phase. The cooling curve of sample G (that achieved undercooling of 611 °C) also shows some sudden jump in the cooling curve (in between T_hyp_ and T_g_). However, in this case, the release of latent heat of crystallization was strong enough to raise the temperature of the molten melt to levels that are higher than the level achieved by the molten droplet just before the recalescence event. This implies that some part of the sample G got converted into crystalline phase, which has been ascertained using XRD profile. Figure 8 shows the time-sequence of the high-speed camera images wherein the temporal history of the solidification phenomenon of samples B, E and F can be seen. Time-sequence images recorded using a high-speed camera after triggering a thin wire of molybdenum where partly glassy and partly crystalline phases can be observed by looking at the bright spot (due to recalescence) and dark zones (glass formation). The comparison of both image-sequence (Figs. 4 and 8) where temporal evolution can be seen, shows how the process of partial crystallization progresses. Relatively larger zones of crystalline enstatite (as compared to a glassy zone of enstatite seen in Fig. 4) can be visualized from the transient evolution of the process of crystallization (see Fig. 8).

**Figure 9:**
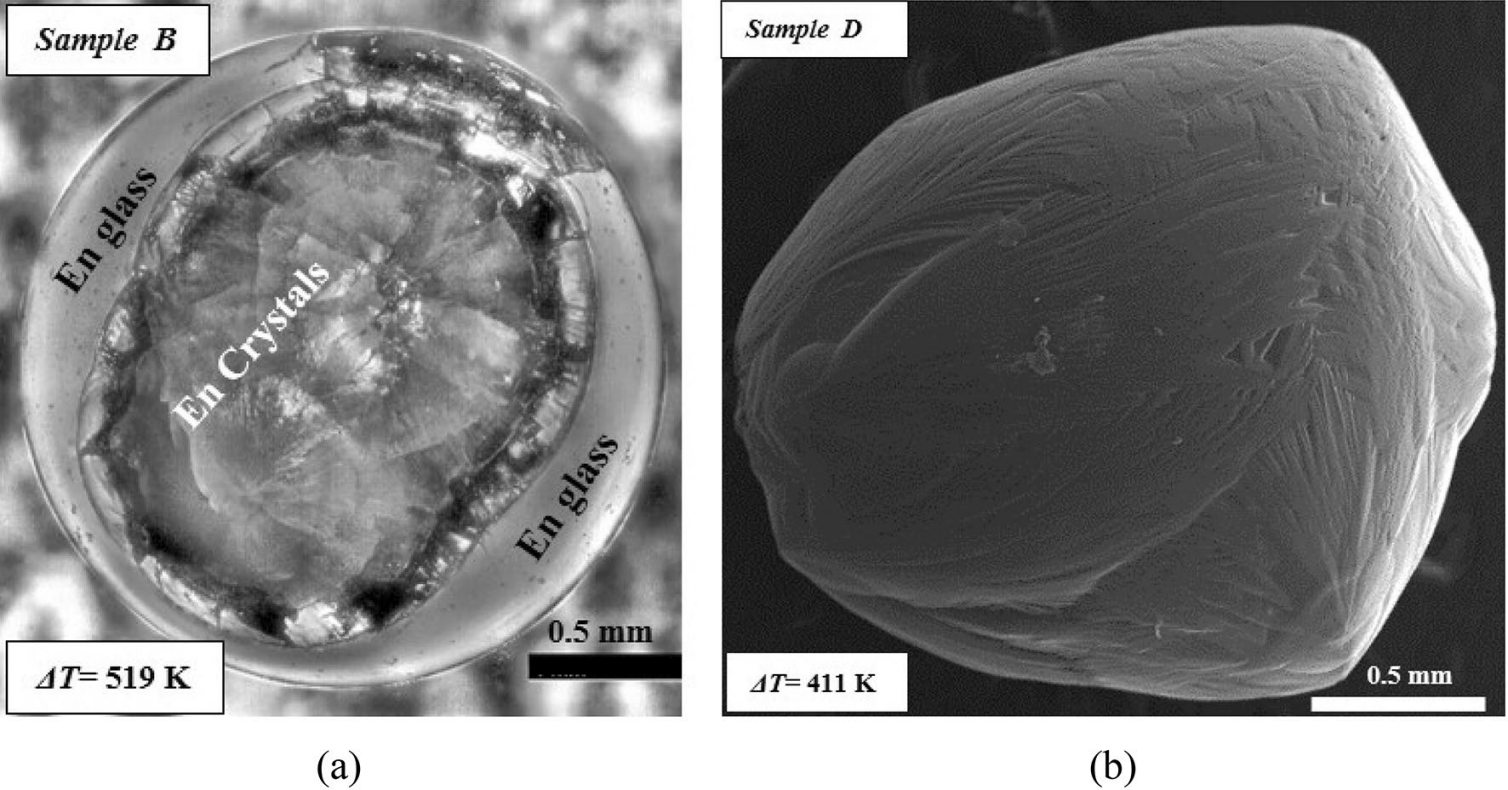
(**a**) Optical microscope image of solidified enstatite sample B (ΔT = 519 K). (**b**) SEM image of solidified enstatite sample D that shows only crystalline region and no glassy region (ΔT = 411 °C).

The above discussion, which is based on the observations from the thermal histories has been extended to surface feature analysis and XRD-based crystalline phase analysis (Fig. 11a). The larger volume of enstatite crystalline region (Fig. 9a) than enstatite glassy region for sample B (as compared to sample A and C) is attributed to the fact that the second recalescence event as shown in the thermal history of sample B is farther from the glass transition line (just near to T_hyp_). While for sample A, this second recalescence event is nearer to the glass transition line (Fig. 3). This may be the possible reason that sample B got more crystallized as compared to sample A where a glassy colony is larger.

Figure 9b shows the SEM image of sample D which was seen to be completely crystallized and no transparent glassy part was to be seen unlike in the case of partially crystallized samples A, B and C. It is to be noted here that for samples A, B and C, optical micrographs rather than SEM images have been shown while for sample D, the discussion is based on SEM image. This approach has been followed in view of the fact that the glassy parts of the partially crystallized enstatite samples (in A, B and C) led to the unavoidable charge-up effects while sample D showed good high-resolution imaging of surface features of the enstatite sample using SEM. XRD scan confirms the crystallinity of sample D where some major and few minor peaks are to be observed (Fig. 11a).

**Figure 10:**
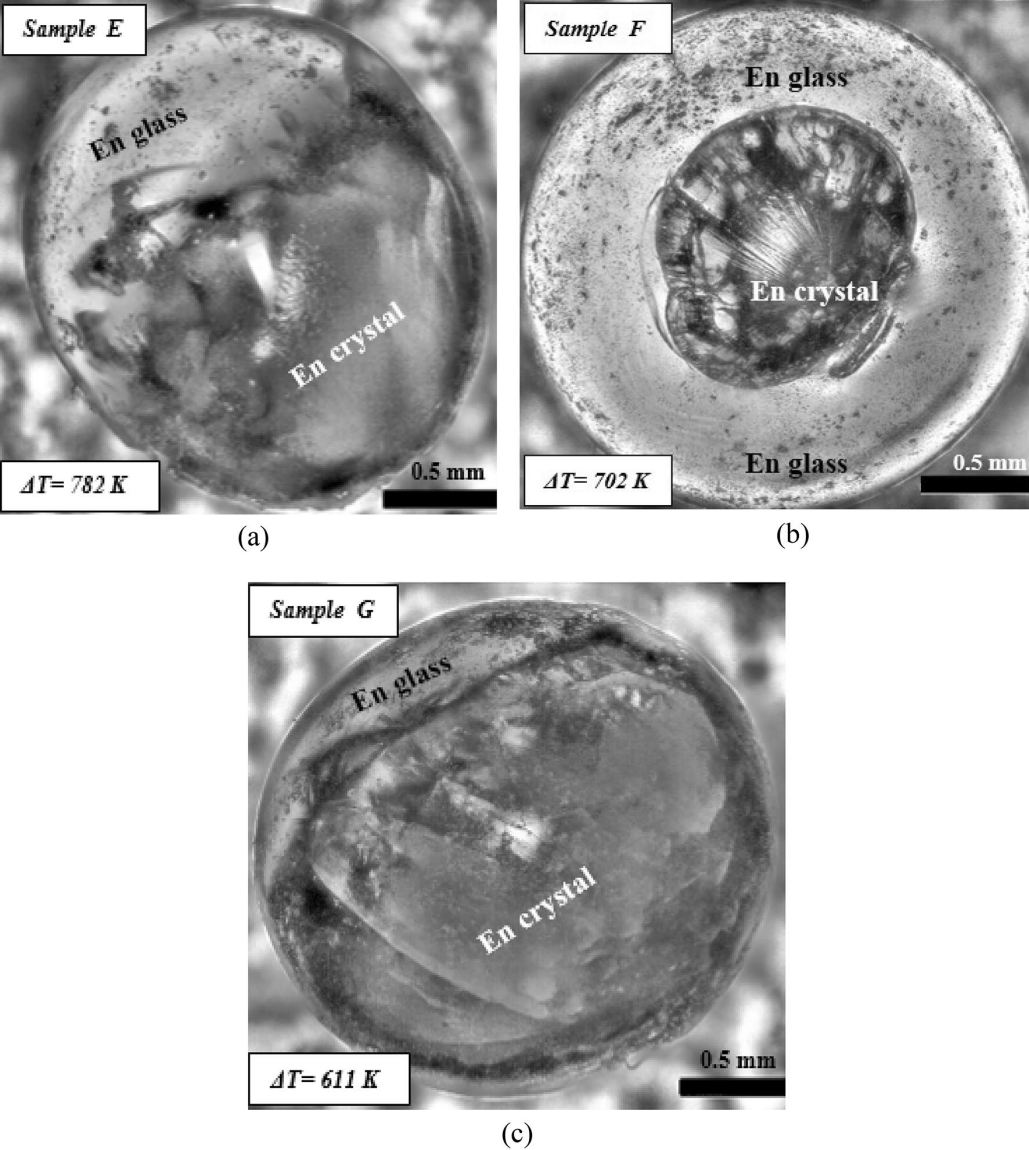
(**a**) Optical microscope image of solidified enstatite sample E, (ΔT = 782 K), (**b**) sample F (ΔT = 702 K) and (**c**) sample G (ΔT = 611 K) processed under levitated conditions and triggered by a wire of molybdenum.

XRD analysis performed on the surface of sample E did not show any peak as was observed in the other samples (sample A, B, C and D), which were partially crystallized, instead showed a halo profile at the start. In order to understand the internal crystallization behaviour of sample E as compared to the surface crystallization where no crystallization was detected from XRD scan of the surface of this sample (E), all the enstatite spherules have been first crushed in an agate mortar to their powdered form and then subjected to XRD analysis. (This aspect of the work has been elaborated in “XRD performed on powdered form of all samples” section). The optical image for sample E (Fig. 10a) showed a glassy region of enstatite (En glass) and crystalline enstatite (En crystal).

**Figure 11:**
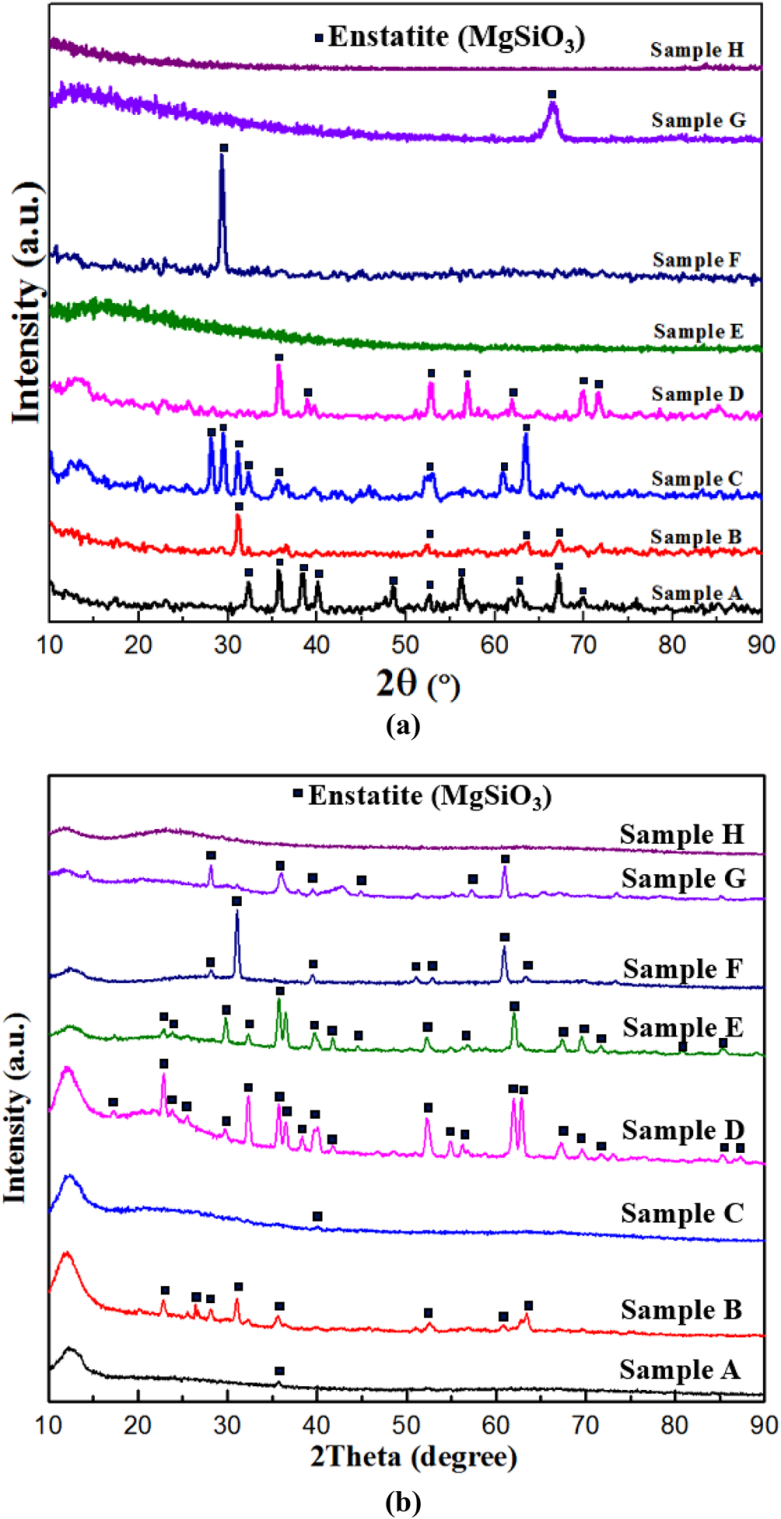
(**a**) XRD patterns obtained from the surface of all samples where samples A, B, C, D, F and G show multiple sharp peaks. However, samples E and H do not show any peaks. (**b**) XRD patterns of the powdered form of all the samples where samples B, D, E, F and G show multiple sharp peaks. Samples A and C exhibit a single and weak peak that suggests detectable crystallization has taken place. However, sample H does not show any peak but shows an amorphous halo in the micrographs.

Figure 10b confirms that a very small amount of crystalline part for sample F is observed while the rest of the part remains glassy. From the XRD profile (Fig. 11a), only a single XRD peak can be seen while other peaks were absent or were beyond the limit of detection. The optical image for sample G shows a small portion of enstatite glass, and a relatively large portion of crystalline enstatite (Fig. 10c). However, the XRD profile exhibits only one sharp peak of enstatite (MgSiO_3_) at 66.7° with a halo pattern nearly at 13° (Fig. 11a).

### XRD analysis

The crystallinity of these spherules was ascertained by carrying out XRD analysis and was confirmed to be of enstatite composition while other phases were below the limit of detection or absent. Two different methodologies of XRD analysis have been adapted to understand the phenomenon of internal as well as surface partial crystallization and are well elaborated in the following sub sections.

#### XRD performed on the crystallized surface of all samples

Figure 11a shows the XRD profiles performed on a crystalline surface of all the samples considered in the present work in which partial crystallization and vitrification process took place. A combination of major and minor peaks for samples A, B, C and D displays that these samples exhibit crystallinity. Also, sample E does not show any XRD peak that does indicate that surface crystallization (as that of all the other samples) is not observed at least for sample E. A single XRD peak has been observed for sample F and sample G. It is pertinent to note here that the possibility of sample evaporation can effectively be ruled out since the final composition obtained from the current XRD data analysis, is the same as the starting material (enstatite (MgSiO_3_)) and hence no extra phase can be seen. Sample H gets converted into a glass and hence its XRD profile shows an initial halo and does not show any major and/or minor peaks.

#### XRD performed on powdered form of all samples

To verify the crystallinity of the inner zones of these samples, XRD analysis has been performed on the powdered form of enstatite spherules (Fig. 11b). XRD scans on the powdered specimens exhibit sharp peaks as well as halo profile at low angle for these spherules, which indicate that crystalline and glassy regions are present in the partially crystallized enstatite spherule. The XRD peak intensity is different for powdered and bulk (solidified) samples. For bulk samples, the XRD peaks are observed only from the focused surface/subsurface, whereas powdered sample gives overall sample information and peak intensity changes accordingly. For example, the powdered form of sample E does show some peaks (Fig. 11b) but an XRD scan performed on the sample surface (Fig. 11a) doesn’t show any peaks. This implies that the spherule has got partially crystallized*.*

Similarly, XRD analysis performed on the crystallized surface of samples A and C showed sharp peaks (Fig. 11a) but that performed on their powdered form showed only a single but weak peak (Fig. 11b). This observation may be attributed to very little crystallization in the sample and the fraction of the glassy region being relatively very large, particularly for these two samples (Samples A and C). This can be well observed in the optical images obtained using the optical microscopy technique (Fig. 5a,b). Also, interaction volume which is subjected is XRD analysis is also constant for all the powdered samples hence such a small fraction of crystalline enstatite spherules (particularly samples A and C) yield in low-intensity peak.

One of the interesting observations made through the present set of experiments revealed that the very thin wire (150 µm) is not sufficient to fully crystallize the levitating and rapidly supercooled enstatite molten droplet. The thin wire that has been brought into contact with the heated surface of the supercooled enstatite spherule offered an extremely lesser area of contact as compared to similar methods employed by some of the previous researchers in the past for initiating heterogeneous nucleation of highly undercooled enstatite spherules. For instance, Nagashima^32^ performed crystallization experiments and hung enstatite spherules using two methods namely, (a) thin PtRh wire loop (0.1 mm diameter), and (b) two parallel PtRh rods of 1 mm thickness. The area of contact was evidently smaller in the case of the wire loop technique as compared to parallel rods and thus influenced the temperature distribution by creating cold spots at the contact points. Moreover, the amount of silica content present in the model material (enstatite) also offers additional resistance to crystallization at deeper undercooling levels and more so when the initially molten sample is subjected to very high cooling rates. These factors lead to vitrification of most of the material, an inference that has been drawn on the basis of the experiments conducted as part of the present work.

## Conclusions

Experimental investigations into the plausible effect of thermal parameters such as levels of undercooling, cooling rate and amount of recalescence on the process of partial crystallization for magnesium silicate material (enstatite spherules as the model material) under purely non-contact conditions were reported. This study suggested that it is insufficient to take into consideration only one single parameter to study the complex mechanism of crystallization. Also, it was the first time that crystallization, which occurs concomitantly with glass formation, has been observed and reported for rapidly cooled enstatite melt under aerodynamically levitated conditions. Optical microscopy and SEM technique have been employed to observe finally solidified structures while XRD analysis confirms the crystallinity of enstatite spherules. Experimental findings proposed the existence of internal and surface crystallization of solidified enstatite spherules. The nature of partial crystallization strongly suggests that enstatite above and below its hypercooling limit (ΔT = 507 °C) exhibits sufficient molecular mobility to support crystallization and hence enstatite spherule shows both crystalline and glassy phases under levitated conditions which have never been explored before.The impact of the thermal history on partial crystallization may provide a pathway, under certain circumstances, to decipher the complex mechanism of formation of the early solar system and shed new light on glass-forming ability of enstatite (magnesium silicate) near and below hypercooling limit. For instance, a recent study of complex forsterite (magnesium silicate) morphologies from dendritic faceted to unbranched faceted features (Shete et al*.*^29^) indicates that such features are affected by undercooling levels.Further work is underway to extend the current study in a more detailed manner to explore the mechanisms of nucleation beyond the hypercooling limit and study the suitability of enstatite samples for mechanical, optical and biomedical applications.
